# Cardiac allograft vasculopathy in Dutch heart transplant recipients

**DOI:** 10.1007/s12471-016-0881-z

**Published:** 2016-09-01

**Authors:** G. Galli, K. Caliskan, A. H. M. M. Balk, R. van Domburg, O. Birim, J. Salerno-Uriarte, O. C. Manintveld, A. A. Constantinescu

**Affiliations:** 1 Department of Cardiology, Erasmus MC, Rotterdam, The Netherlands; 2 Department of Cardiology, University of Insubria, Ospedale di Circolo and Fond. Macchi, Varese, Italy; 3 Department of Cardiothoracic Surgery, Erasmus MC, Rotterdam, The Netherlands

**Keywords:** Cardiac allograft vasculopathy (CAV), Donor age, Heart transplantation prognosis

## Abstract

**Background:**

Cardiac allograft vasculopathy (CAV) is a multifactorial disease and a major cause of graft failure after heart transplantation. However, the impact of CAV may vary according to the definition and the regional differences in transplantation settings.

**Objectives:**

We sought to assess CAV prevalence, predictors and prognosis in Dutch heart transplant recipients based on coronary angiography, following the 2010 standard nomenclature of the International Society for Heart and Lung Transplantation.

**Methods:**

Patients ≥18 years who underwent heart transplantation at our centre with at least one coronary angiography during follow-up were included in the analysis. Clinical variables were collected prospectively.

**Results:**

Among 495 analysed recipients, there were 238 (48 %) with CAV. The prevalence of CAV was 18, 47 and 70 % at 4, 12 and 20 years, respectively. In the multivariable proportional hazards regression analysis, only male donor gender and increasing donor age were significantly associated with the risk of CAV. The long-term prognosis of the patients with CAV at fourth-year angiography was significantly worse as compared with that of CAV-free patients, independently of the severity of CAV (*p* < 0.001).

**Conclusion:**

The prevalence of CAV increased gradually over time, with a similar trend as in other registries. Post-transplant survival is decreased in patients with any degree of early CAV, indicating that management strategies should start with donor selection and preventive measures immediately after transplantation.

## Introduction

Cardiac allograft vasculopathy (CAV) is one of the major causes of late graft failure and death in heart transplant patients [1]. The reported CAV prevalence varies according to the definition, population, transplantation period and follow-up protocol and ranges from 39 to 65 % at 10 years in single-centre studies, while in the large register of the International Society for Heart and Lung Transplantation (ISHLT) it is 50 % at 10 years [1–3]. CAV is characterised by concentric thickening of the wall of large and small coronary vessels and has various histological patterns, including inflammatory lesions, lesions rich in smooth muscle cells and fibrotic lesions, which have been related to the time passing after transplantation [4, 5]. The pathogenesis of CAV has been related to immunological and non-immunological factors in both the donor and the recipient, but the exact triggers and the pathophysiological pathways are still unknown [6, 7]. The data are heterogeneous due to different transplantation decades, different populations and treatment protocols and their generalisability is further hampered by various diagnostic criteria of CAV [6, 8]. Standardisation of the CAV diagnosis and gradation was recommended in 2010 by the ISHLT based on conventional coronary angiography [9].

In the Netherlands, the shortage of the donors has led to an increase in the mean donor age from 29 to 43 years, while the most frequent cause of death shifted from trauma to stroke. Despite the use of older donors, we found an improved survival after heart transplantation in the last decade at our centre [10]. However, subclinical atherosclerosis may be more frequent in donor hearts from older patients with neurovascular comorbidity and, therefore, the first aim of the current study was to investigate CAV prevalence and predictors in the patients undergoing heart transplantation in the Netherlands, using our large single-centre cohort. Secondly, we aimed to assess the long-term prognosis taking into account the diagnosis and severity of CAV.

## Patients and methods

### Study population

Since the first orthotopic heart transplantation at our centre in June 1984, data of all heart transplant recipients were collected prospectively until December 2012. Patients consented to the use of anonymised data for research purposes. The institutional review board of the Erasmus MC approved the present study.

Only patients ≥18 years who underwent at least one conventional coronary angiography at follow-up were included in the analysis. We recorded recipient-related and donor-related variables based on the clinical relevance and previously published studies on CAV predictors. Recipient pre-transplant clinical variables were age, gender, aetiology of heart failure, creatinine and diabetes. Donor-related data were age, gender and cause of death. Donor-recipient mismatch variables and available immunological information were collected. Data at one year after transplantation included the number of acute rejection episodes, development of cytomegalovirus-related disease, serum creatinine, total cholesterol, triglycerides, diagnosis of hypertension and diabetes. Rejection surveillance was based on endomyocardial biopsies, which were graded according to the Billingham’s criteria until 2004 [11, 12] and subsequently according to the ISHLT revised guidelines [13]. Acute rejections were defined as the treated rejections within the first year after transplantation in each patient. Immunosuppressive medication and the use of statins were recorded at the time of CAV diagnosis or at the time of the most recent angiography for the patients without CAV. Mortality and retransplantation were recorded as outcome events.

### Diagnosis of CAV

Coronary angiography (CAG) was performed per protocol every year until 1990. After 1990, CAG was performed at one year and repeated every year in case of evidence of CAV, otherwise postponed to the fourth year after transplantation [14]. After the fourth year post-transplantation, patients underwent non-invasive myocardial perfusion scintigraphy annually for assessment of ischaemia. CAG was performed in these patients when perfusion scintigraphy was positive, or when ischaemia was suspected based on cardiac markers or clinical, electrocardiographic or echocardiographic criteria. Two cardiologists reviewed all the available coronary angiographies, or, when the CAG images were absent in our archive, the diagnosis of CAV was made from the written report and re-graded according to the 2010 ISHLT criteria [9]. In summary, CAV grade 1 applies to stenosis <70 % of primary or secondary coronary branches, CAV grade 2 applies to stenosis ≥70 % of one primary branch or two secondary branches, CAV grade 3 applies to stenosis ≥70 % of two primary branches or three secondary branches, or if an impaired left ventricular function is associated with any degree of CAV. For each patient we recorded the time from heart transplant until the first CAG showing any degree of CAV.

## Statistical analysis

For the analysis of CAV prevalence, the proportion of patients with CAV among survivors was calculated at predefined points, i. e. at 1, 4, 8, 12, 16 and 20 years post-transplantation. Donor and recipient variables were compared between CAV-free and CAV patients. Categorical data are presented as numbers with percentages. Continuous data are presented as mean ± standard deviation. The data were compared using the chi-square test for categorical variables, and independent sample t‑test for continuous variables. Univariate analysis of donor and recipient characteristics was performed using the Cox proportional hazards model. Significant results from the univariate analysis (*p* ≤ 0.05), but also non-significant clinically relevant variables were included in the multivariable proportional hazards regression analysis using the backward procedure. For survival analysis, the outcome was the combination of all-cause mortality and retransplantation according to the presence or absence of CAV using the Kaplan-Meier method and the log-rank test for comparison. Data were analysed using IBM SPSS Statistics version 21.

## Results

**Fig. 1:**
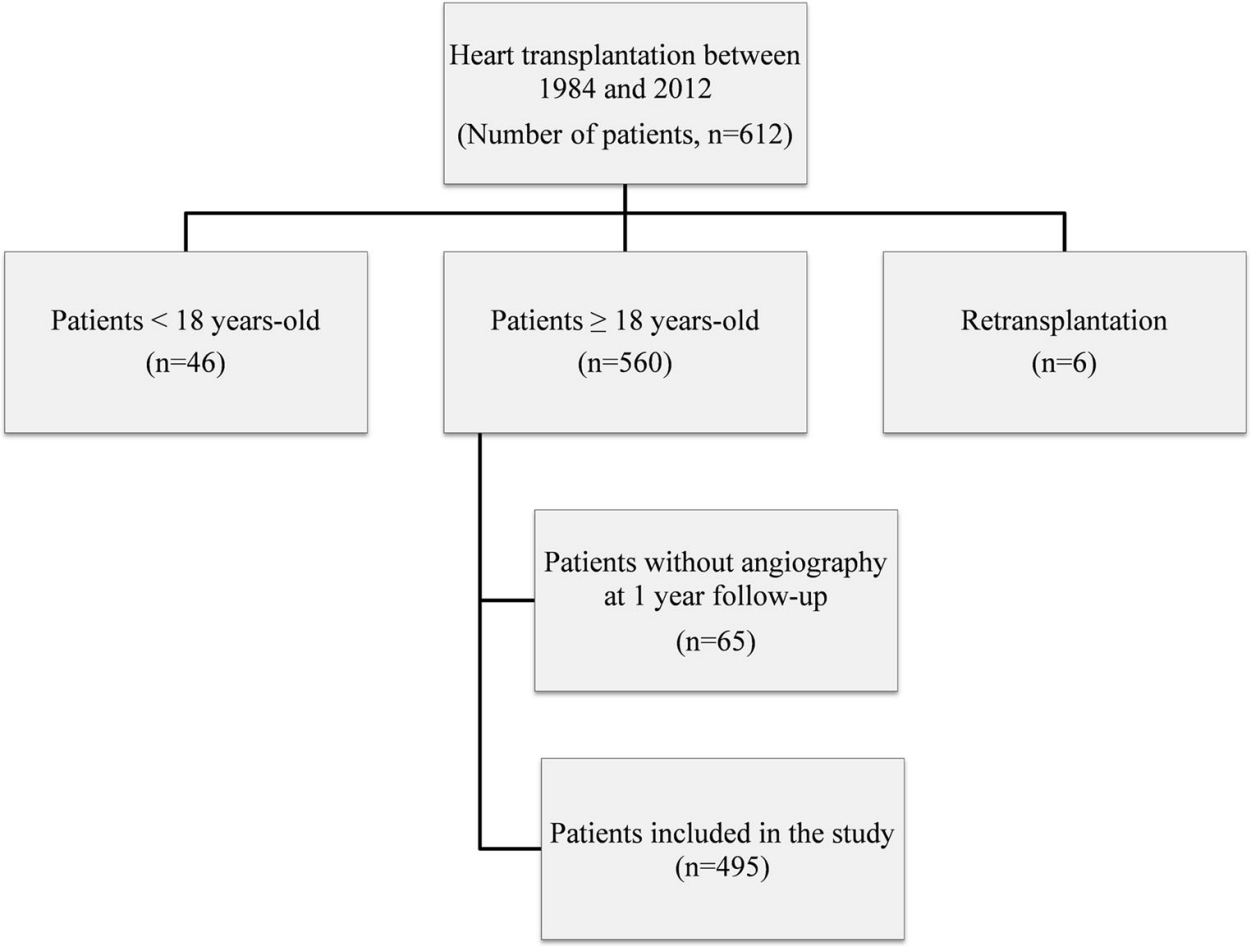
Flowchart study population

From the cohort of 612 heart transplant recipients, 495 were included in the analysis (Fig. 1). The mean age at transplantation was 48.6 ± 10.3 years, and 77.4 % of recipients were males. Ischaemic heart disease was the cause of heart failure before transplantation in 49.9 % of the patients. Diabetes was present in 6.1 % of the patients before transplantation. Mean donor age was 33.3 ± 12.9 years and 52.7 % of the donors were males. The cause of death was trauma in 48.8 % of the donors. During the first year post-transplantation, hypertension, diabetes and cytomegalovirus disease were present in 75, 32 and 20 % of the patients, respectively. At one year after transplantation only a minority of the recipients (22.6 %) were free of any rejection episodes, while 21.6 % had more than two rejection episodes.

**Fig. 2:**
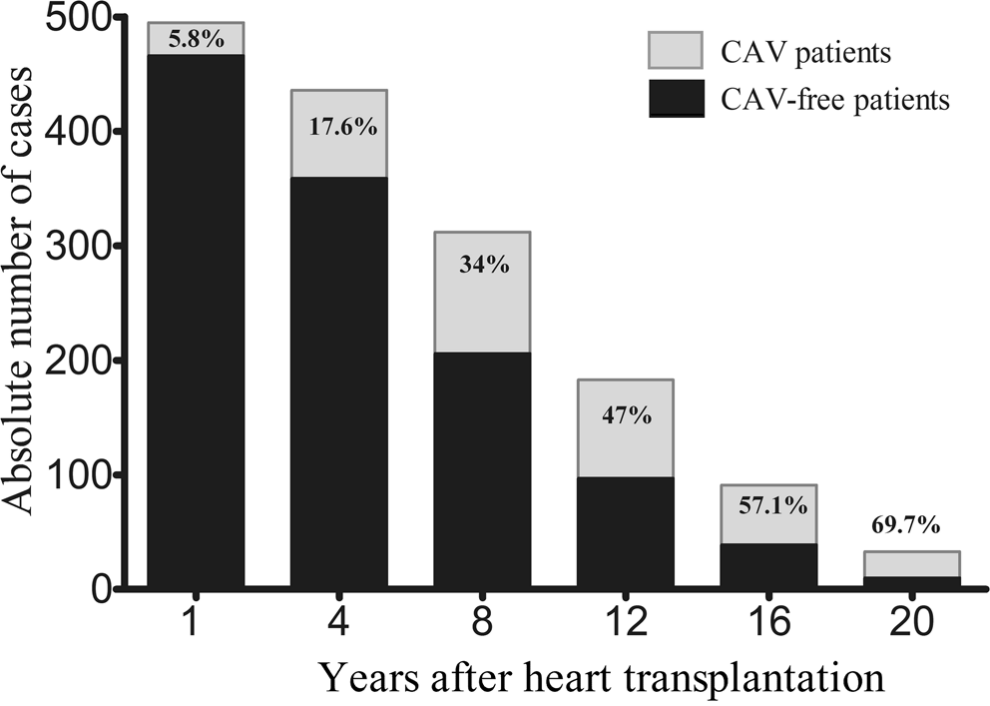
CAV prevalence in survivors after heart transplantation

A total of 238 (48.1 %) transplant recipients received the diagnosis of CAV. Mean time to CAV detection was 6.1 ± 4.2 years after transplant, with the median at the fourth year after transplantation, when CAG was routinely performed according to the follow-up protocol. The majority of the affected patients (60.1 %) had a mild CAV (grade 1), while 12.6 % of the patients had severe CAV (grade 3) at diagnosis (Table 1). The prevalence of CAV increased gradually from 17.6 % at 4 years to 47 % at 12 years and 69.7 % at 20 years, while the number of surviving transplant recipients decreased during the follow-up period (Fig. 2).

**Table 1:** Detection of CAV after heart transplantation

	***N*** ** (%)**
Mean follow-up	10.6 ± 5.7 years
*N*. patients with CAV diagnosis	238 (48.1)
Time to CAV after heart transplantation Median Range	6.1 ± 4.2 years 4.9 years 17 days–18 years
CAV grade at diagnosis 1 2 3	– 143 (60.1) 65 (27.3) 30 (12.6)

The recipient characteristics before and after transplantation, donor characteristics, immunosuppression and statin therapy were compared between the cohorts of patients with CAV and without CAV (Table 2). a significant difference in the recipient age at transplantation and the donor age was seen between the two cohorts. There was a significantly higher proportion of male donors and higher cholesterol levels at one year in the CAV cohort as compared with the CAV-free cohort. No significant difference was found in the immunosuppression induction therapy or maintenance immunosuppression or statins therapy at the moment of CAV diagnosis, renal function or the presence of diabetes between the two cohorts. There was a trend for a larger proportion of patients without any rejection in the CAV-free cohort as compared with the CAV cohort (*p* = 0.06).

**Table 2:** Donor and recipient variables in CAV-free and CAV-affected patients

	CAV-free population, Group I (*n* = 257), *N* (%)	CAV population, Group II (*n* = 238), *N* (%)	*p* value^a^
*Recipient*			
Recipient age, years 18–39 40–49 50–59 ≥60	49.7 ± 10.2 40 (15.6) 74 (28.8) 105 (40.9) 38 (14.8)	47.4 ± 10.3 46 (19.3) 74 (31.1) 97 (40.8) 21 (8.8)	0.01 0.18
Recipient gender: Male/Female	192/65 (74.7/25.3)	191/47 (80/19.7)	0.11
Previous ischaemic heart disease	120 (46.7)	127 (53.4)	0.14
Creatinine pre-heart transplantation (µmol/l)	121.2 ± 42.9	121.7 ± 51.6	0.92
Diabetes pre-heart transplantation	15 (5.8)	15 (6.4)	0.81
*Donor*			
Donor age, years	32.1 ± 13.4	34.7 ± 12.3	0.03
12–29 30–39 40–49 ≥50	122 (48.2) 54 (21.3) 43 (17) 34 (13.4)	89 (37.9) 58 (24.7) 59 (25.1) 29 (12.3)	0.06
Donor death for head trauma	110 (47.4)	105 (50.2)	0.53
Donor gender: male/female	121/134 (47.5/52.5)	140/96 (59.3/40.7)	0.01
*Mismatches donor/recipient*			
Gender mismatch: donor/recipient	99 (38.8)	83 (35.2)	0.4
Cytomegalovirus serology mismatch	59 (23)	55 (23.1)	0.97
Toxoplasmosis serology mismatch	41 (16)	41 (17.2)	0.7
HLA antibodies mismatch	69 (28.2)	73 (31.9)	0.38
*Surgery and medical therapy*			
Ischaemia time	170.5 ± 42.1	176.6 ± 45.7	0.12
Cyclosporine	145 (56.4)	147 (61.8)	0.23
Tacrolimus	112 (43.6)	89 (37.4)	0.16
Mycophenolate mofetil (plus tacrolimus)	65 (25.3)	65 (27.3)	0.61
Everolimus (plus tacrolimus)	7(2.7)	10 (4.2)	0.37
Statin	114 (44.5)	112 (47.1)	0.55
Immunosuppressive induction therapy	208 (80.9)	181 (76.1)	0.19
*Parameters at one year after heart transplantation*			
Cholesterol (mmol/l)	6.4 ± 1.7	6.7 ±2	0.03
Triglycerides (mmol/l)	2.3 ± 1.1	2.3 ±1	0.89
Hypertension	190 (73.9)	181 (76.1)	0.59
Diabetes	84 (32.7)	76 (32.1)	0.88
Creatinine (µmol/l)	144.5 ± 79.6	135.4 ± 59.1	0.14
Cytomegalovirus disease (within first year)	45 (17.5)	55 (23.1)	0.12
Absence of rejection episodes	67 (26.1)	45 (18.9)	0.06
Total number of rejection episodes	1.7 ± 1.6	1.9 ± 1.6	0.13

All results are expressed as absolute numbers and percentages or mean ± standard deviation. *P*-values were obtained with Pearson’s chi-square test or t‑test for equality of means. *HLA* human leucocyte antigen

**Fig. 3:**
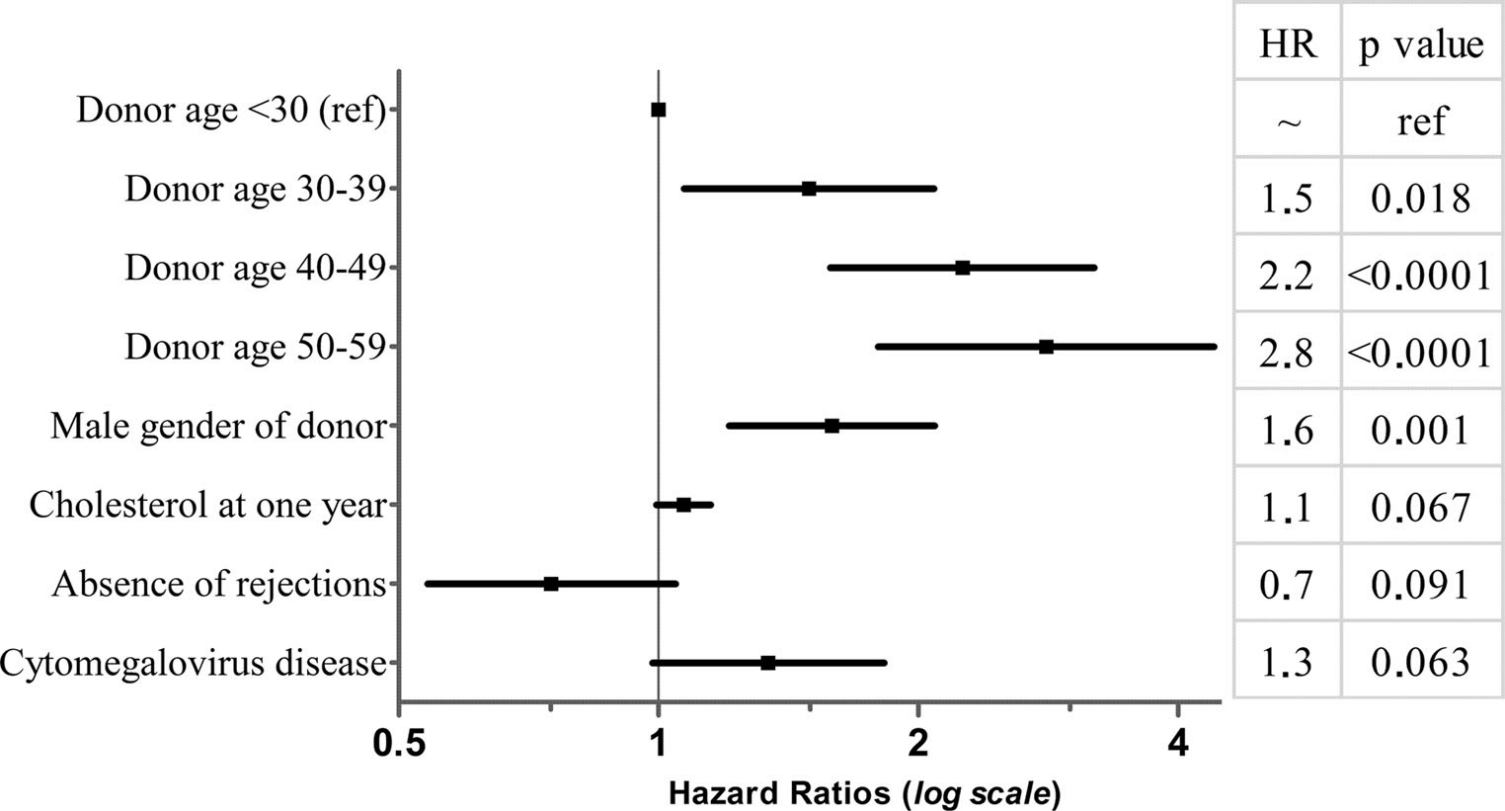
Forest plot of risk factors included in the multivariate Cox proportional hazard modelling

In the multivariable proportional hazard regression analysis only male donor gender and donor age were significantly associated with CAV, with a gradual increased hazard risk of 1.5, 2.2 and 2.8 in the three consecutive groups of donor age, while post-transplantation clinical factors were not significant (Fig. 3). The treatment with mycophenolate mofetil was associated with a significantly increased risk (HR 1.4, *p* = 0.008) only in the univariate analysis, while cytomegalovirus disease was associated with an increased hazard risk for CAV (HR 1.3, *p* = 0.059), although not significant, in the univariate analysis (Table 3).

**Table 3:** Univariate Cox regression analysis of determinants of CAV

	HR	95 % CI	*p* value
*Donor age (ref. age <40)*			
40–49	1.4	1.04 ± 2.01	0.03
50–59	2	1.40 ± 2.72	<0.0001
>60	2.2	1.45 ± 3.39	<0.0001
*Recipient age (ref. age 18–30)*			
30–39	1.1	0.73 ± 1.52	0.79
40–49	1.1	0.76 ± 1.53	0.68
≥50	0.8	0.48 ± 1.35	0.42
Male gender of donor	1.3	0.97 ± 1.63	0.09
Male gender of recipient	1.2	0.89 ± 1.68	0.23
Donor death for trauma	1.2	0.92 ± 1.58	0.18
Previous ischaemic heart disease	1.3	0.98 ± 1.64	0.07
Creatinine pre-heart transplantation	1.0	1.00 ± 1.00	0.82
Diabetes pre-heart transplantation	1.3	0.75 ± 2.13	0.39
Ischaemia time	1	1.00 ± 1.00	0.77
Toxoplasmosis serology mismatch	1.1	0.82 ± 1.61	0.42
Cytomegalovirus serology mismatch	1.1	0.78 ± 1.42	0.74
Cytomegalovirus disease	1.3	0.99 ± 1.81	0.059
Absence of any episodes of rejection	0.8	0.57 ± 1.09	0.16
HLA antibodies mismatch	0.9	0.68 ± 1.19	0.47
Induction treatment	1.1	0.83 ± 1.51	0.47
Cyclosporine	0.9	0.69 ± 1.16	0.4
Tacrolimus	1.1	0.83 ± 1.41	0.55
Mycophenolate mofetil	1.4	1.11 ± 1.97	0.008
Everolimus	1.5	0.82 ± 2.20	0.18
Statin	1.1	0.82 ± 1.37	0.64
Triglycerides (at one year)	1	0.86 ± 1.09	0.6
Cholesterol (at one year)	1	0.96 ± 1.10	0.48
Diabetes (at one year)	0.9	0.72 ± 1.24	0.68
Creatinine (at one year)	1	1.00 ± 1.00	0.82
Hypertension (at one year)	0.9	0.65 ± 1.19	0.4

*HLA* human leucocyte antigen

**Fig. 4:**
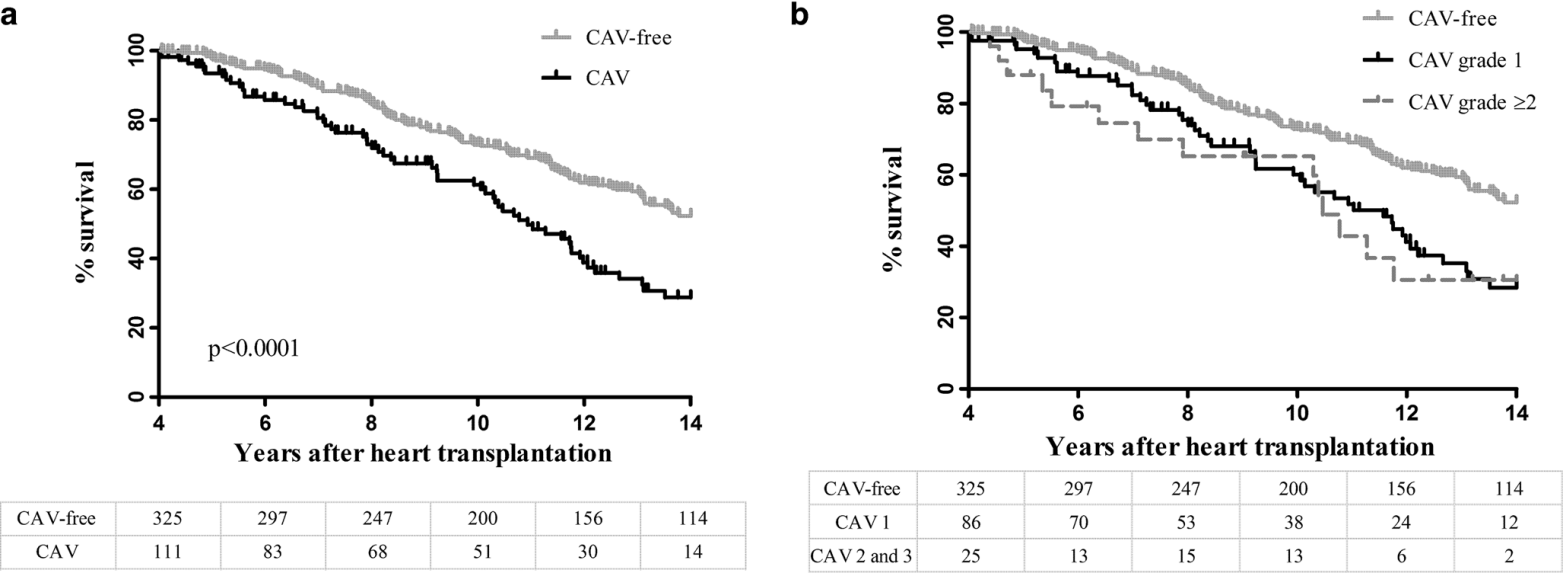
**a** Long-term survival in CAV versus CAV-free patients. Kaplan-Meier analysis used to compare survival of patients alive at four years, grouped into patients with CAV diagnosis within the fourth year and CAV-free patients. Curves were compared with log-rank test. **b** Long-term survival according to CAV severity at four years. Log-rank overall: p < 0.0001, while log-rank analysis between CAV1 versus CAV ≥2 was not significant (p = 0.0656)

The long-term outcome consisting of survival or retransplantation was compared between the patients with CAV of any degree at fourth-year angiography and the patients with no CAV at the same evaluation (Fig. 4a). The patients with CAV diagnosis had a significantly impaired prognosis as compared with CAV-free patients at the fourth year after transplantation (*p* < 0.001). Also, significantly decreased long-term survival was found for the patients with mild CAV (grade 1) when compared with CAV-free patients and there was no difference in survival of the CAV grade 1 group when compared with more advanced grades of CAV (Fig. 4b).

## Discussion

Our study has several findings: 1) the prevalence of CAV increased gradually during follow-up to 70 % of the survivors at 20 years; 2) donor-related factors were associated with an increased risk of CAV and 3) all CAV severity grades, including mild coronary lesions, at the fourth year after heart transplantation, are associated with a decreased long-term survival.

### Prevalence and predictors of CAV

Although the donors in the Netherlands may be older than in other transplantation settings, CAV prevalence in our cohort was comparable with that presented in the most recent report of the ISHLT [1]. However, the strongest CAV risk factor in our study was older donor age. Donor age has been previously reported to be associated with an increased risk of CAV in the database of the United Network for Organ Sharing (in the United States), as well as in Spanish heart transplant registries [15, 16]. We found that also male donor sex was associated with an increased risk of CAV, irrespective of the gender of the recipient. Other studies have shown that gender mismatch, particularly a male recipient receiving a female donor heart, was associated with decreased survival after transplantation, but the reports are contradictory about its relationship with the risk the CAV [17]. Although we did not collect data on donor risk factors, older and male donors might reasonably have higher rates of hypertension, dyslipidaemia and/or history of smoking, conferring the higher chance of subclinical vascular atherosclerosis that might be correlated to neo-intima fibrosis in CAV. Furthermore, donor-derived immunological factors have been reported to contribute to the development of CAV in addition of recipient immunity [18].

We could not find any recipient-related independent predictors of CAV, although in the univariate analysis the risk of CAV was significantly associated with the treatment with mycophenolate mofetil, while cytomegalovirus disease had a trend towards positive association with CAV. The role of cytomegalovirus disease in the development of CAV has been extensively studied, and a positive association has not always been found, but a recent Spanish report has shown that cytomegalovirus disease and even asymptomatic viraemia are associated with increased risk [3]. The possible explanations for the association between treatment with mycophenolate mofetil and CAV in the univariate analysis, but not in the multivariable analysis, could be an increased susceptibility for cytomegalovirus disease during mycophenolate mofetil treatment, or the need for increased immunosuppression due to rejection episodes. Other studies have shown that high rejection scores or repeated acute cardiac rejections are independent predictors of CAV [7, 19]. We did not find a significant association after Cox regression analysis, although the number of patients without any rejection episodes was higher in the CAV-free cohort as compared with the CAV cohort. No other transplantation treatment was found to be associated with CAV. The use of statins has been associated with increased survival after heart transplantation, and it is recommended in the guidelines for the care of heart transplant recipients [20]. One possible explanation for the lack of a positive effect can be the inclusion of patients transplanted before the statin era in the analysis. The number of patients treated with everolimus, as maintenance therapy before coronary angiography, was too low to assess its association with CAV.

### Prognosis of CAV

We present the survival curves according to the presence of CAV at four years after transplantation, because all patients then underwent elective coronary angiography, according to the protocol at our centre. After the fourth year, patients underwent myocardial perfusion scintigraphy yearly for evaluation of ischaemia, and coronary angiography was only performed by indication. An important finding of our study was that not only is early CAV diagnosis at four years associated with decreased long-time survival, but also that even mild coronary lesions, scored as CAV grade 1, represent a negative prognostic factor. Although the majority of studies are directed to more advanced degrees of CAV, which are also amenable to interventions, one other recent report showed that persistent mild coronary lesions have a negative prognostic impact [2, 21]. This finding emphasises the role of preventive measures immediately after transplantation, before even mild coronary disease may develop.

## Limitations of the study

This is a retrospective study, although based on a prospective cohort. The multivariate analysis of the clinical factors allows only the finding of associations with CAV, but not causal relations. Other variables potentially of interest may not have been prospectively collected or analysed. The study is limited to a single centre, and therefore other centre-related factors may prevent the generalisation of the findings.

## Conclusion

Our study provides an overview of CAV prevalence and predictors in the heart transplant recipients in the Netherlands. The novel finding is that early CAV grade 1, representing mild disease, is associated with decreased long-term graft survival. We conclude that the management of CAV should start with donor selection and with preventive medical treatment according to the guidelines of transplant care, immediately after heart transplantation.
